# CRISPR/Cas9‐mediated targeted mutagenesis of *GmFT2a* delays flowering time in soya bean

**DOI:** 10.1111/pbi.12758

**Published:** 2017-06-20

**Authors:** Yupeng Cai, Li Chen, Xiujie Liu, Chen Guo, Shi Sun, Cunxiang Wu, Bingjun Jiang, Tianfu Han, Wensheng Hou

**Affiliations:** ^1^ National Center for Transgenic Research in Plants Institute of Crop Sciences Chinese Academy of Agricultural Sciences Beijing China; ^2^ Ministry of Agriculture Key Laboratory of Soybean Biology (Beijing) Institute of Crop Sciences Chinese Academy of Agricultural Sciences Beijing China

**Keywords:** soya bean, *GmFT2a*, *Agrobacterium tumefaciens*‐mediated transformation, CRISPR/Cas9, genome editing, flowering time

## Abstract

Flowering is an indication of the transition from vegetative growth to reproductive growth and has considerable effects on the life cycle of soya bean (*Glycine max*). In this study, we employed the CRISPR/Cas9 system to specifically induce targeted mutagenesis of *GmFT2a*, an integrator in the photoperiod flowering pathway in soya bean. The soya bean cultivar Jack was transformed with three sgRNA/Cas9 vectors targeting different sites of endogenous *GmFT2a* via *Agrobacterium tumefaciens*‐mediated transformation. Site‐directed mutations were observed at all targeted sites by DNA sequencing analysis. T1‐generation soya bean plants homozygous for null alleles of *GmFT2a* frameshift mutated by a 1‐bp insertion or short deletion exhibited late flowering under natural conditions (summer) in Beijing, China (N39°58′, E116°20′). We also found that the targeted mutagenesis was stably heritable in the following T2 generation, and the homozygous *GmFT2a* mutants exhibited late flowering under both long‐day and short‐day conditions. We identified some ‘transgene‐clean’ soya bean plants that were homozygous for null alleles of endogenous *GmFT2a* and without any transgenic element from the T1 and T2 generations. These ‘transgene‐clean’ mutants of *GmFT2a* may provide materials for more in‐depth research of *GmFT2a* functions and the molecular mechanism of photoperiod responses in soya bean. They will also contribute to soya bean breeding and regional introduction.

## Introduction

Soya bean (*Glycine max* (L.) Merr.) is an important legume crop with great economic value that provides abundant protein and oil for food production and animal feed. It is also a short‐day dicotyledon and is sensitive to seasonal changes in day length. This photoperiod sensitivity limits its geographical range of cultivation, and thus investigating the photoperiod response in flowering induction has great significance in soya bean regional introduction and domestication (Wang *et al*., 2016; Xu *et al*., 2013). In recent years, molecular biological studies in *Arabidopsis thaliana* have shown that *FLOWER LOCUS T* (*FT*) encodes florigen and plays an important role in flowering pathways as an integrator (Corbesier and Coupland, 2006; Turck *et al*., 2008). FT protein is a florigen that moves through the phloem to the shoot apex and functions as a long‐distance signal that induces floral initiation of *Arabidopsis* (Corbesier *et al*., 2007; Jaeger and Wigge, 2007; Mathieu *et al*., 2007; Notaguchi *et al*., 2008). Loss of *FT* function in *A. thaliana* results in a late‐flowering phenotype, whereas overexpression of *FT* causes precocious flowering independent of the transcription factor *CONSTANS* (*CO*) or photoperiod (Kobayashi *et al*., 1999; Koornneef *et al*., 1991). In soya bean, some homologous genes of *FT* have similar functions. Ten *FT* homologs in soya bean have been identified, and two, *GmFT2a* (Glyma16g26660) and *GmFT5a* (Glyma16g04830), have been confirmed to have functions similar to those of *FT* based on ectopic expression analysis in *Arabidopsis*. These homologs also coordinately control flowering in soya bean (Kong *et al*., 2010). Analysis of *GmFT2a* transcripts revealed that the expression of *GmFT2a* is regulated by photoperiod and is associated with flowering induction and maintenance (Sun *et al*., 2011). The *GmFT2a* promoter region harbours rich polymorphisms among different soya bean cultivars, but its coding sequence is highly conserved, and the polymorphisms in *GmFT2a* are not responsible for maturity diversity in soya bean (Jiang *et al*., 2013a). Subsequent studies have shown that *GmFT2a* and *GmFT5a* promote early flowering in soya bean upon overexpression of these two genes in the soya bean cultivar Williams 82 under long‐day (LD) conditions (Nan *et al*., 2014). Several flowering‐related genes in soya bean, such as *GmAP1*,* GmSOC1* and *GmLFY*, were significantly up‐regulated by *GmFT2a* and *GmFT5a* on the basis of a redundant and differential pattern. Yeast two‐hybrid and bimolecular fluorescence complementation (BiFC) demonstrated that both *GmFT2a* and *GmFT5a* interact with the bZIP transcription factor *GmFDL19*, which can also cause early flowering (Nan *et al*., 2014). Fine mapping, sequencing and expression analysis revealed that the soya bean maturity gene *E9* is *FT2a*, and that its recessive allele causes late flowering as a result of attenuated transcript abundance induced by allele‐specific transcriptional repression due to the *Ty1*/*copia*‐like retrotransposon *SORE‐1* inserted in the first intron. *SORE‐1* was highly methylated and did not generate damage to *FT2a* RNA processing (Zhao *et al*., 2016a). Obviously, *GmFT2a* plays an important role in flowering induction and maintenance in soya bean. However, previous studies of *GmFT2a* have mainly involved overexpression or ectopic expression analysis, and studies of soya bean endogenous gene *GmFT2a* mutants with loss of gene function remain relatively limited.

The recently developed CRISPR (clustered regularly interspaced short palindromic repeat)/Cas9 (CRISPR‐associated) system has provided a robust and effective tool for targeted genome editing and more choice for gene functional researches. The main characteristic of the CRISPR/Cas9 system is the Cas9 protein, which comprises of two nuclease domains: the RuvC‐like domain and HNH domain (Cong *et al*., 2013). The Cas9 protein can form a complex with a synthetic sgRNA, which guides it to recognize target sequences and generate double‐strand breaks (DSBs) at expected target sites (Jinek *et al*., 2012). The DSBs subsequently induce DNA self‐repair mechanisms in the cell, which mainly include nonhomologous end‐joining (NHEJ) and homology‐directed repair (HDR). The NHEJ pathway is error‐prone and usually introduces some base insertions or deletions (indels) at the DNA break sites (Gorbunova and Levy, 1999). When these indels generate a frameshift mutation or disrupt important functional domains, the functions of the target genes will be damaged (Shan *et al*., 2013). The HDR pathway is a precise DNA repair mechanism that can be utilized to introduce specific point mutations or insertions of desired sequences at the target sites (Shan *et al*., 2013; Svitashev *et al*., 2015). The sequence of a fragment could also be replaced with desired sequences through the HDR pathway in the presence of an exogenous donor template (Gratz *et al*., 2014; Zhao *et al*., 2016b). The earliest reports of CRISPR/Cas9‐mediated genome editing in plants date from 2013 (Jiang *et al*., 2013b; Li *et al*., 2013). This new system has since been successfully applied to generate and estimate genome editing in many major crops, such as rice (Shan *et al*., 2013), wheat (Upadhyay *et al*., 2013), sorghum (Jiang *et al*., 2013b) and maize (Liang *et al*., 2014). CRISPR/Cas9‐mediated genome editing in soya bean was first successfully achieved in 2015. The CRISPR/Cas9 system has been successfully utilized to generate and estimate targeted mutations in both endogenous and exogenous genes in soya bean hairy roots (Cai *et al*., 2015; Du *et al*., 2016; Jacobs *et al*., 2015; Michno *et al*., 2015; Sun *et al*., 2015; Tang *et al*., 2016) and whole plants from embryonic calluses transformed by particle bombardment (Li *et al*., 2015). Targeted gene integrations through HDR were also detected by border‐specific polymerase chain reaction analysis at the callus stage, which revealed that one HDR event was transmitted to the T1 generation (Li *et al*., 2015). *Rj4*, not the gene previously reported, was confirmed to be the gene controlling nodulation specificity in soya bean through both complementation tests and CRISPR/Cas9‐mediated gene knockout experiments (Tang *et al*., 2016). These works have shown that CRISPR/Cas9 is a simple, efficient and highly specific genome editing tool in soya bean, although valuable phenotypic alterations have not been reported.

In this study, we employed the CRISPR/Cas9 system to specifically induce targeted mutagenesis of the *GmFT2a* gene in soya bean. A variety of homozygous *ft2a* mutants were generated from a sufficient number of stable transgenic soya bean events developed via *Agrobacterium*‐mediated transformation. In the results of *GmFT2a*‐CRISPR/Cas9, T1‐generation soya bean plants homozygous for null alleles of *GmFT2a* frameshift mutated by a 1‐bp insertion or short deletions exhibited late flowering under natural conditions (summer) in Beijing, China (N39°58′, E116°20′). The targeted mutations were stably inherited and maintained consistent mutation types from the T1 to T2 generation, and the homozygous T2 *ft2a* mutants exhibited late flowering under both long‐day (LD, 16 h light/8 h dark) and short‐day (SD, 12 h light/12 h dark) conditions. These results may contribute to soya bean regional introduction. The use of the CRISPR/Cas9 system to generate a phenotype of important agronomic traits in soya bean stable transformation has not been reported previously. We also obtained some ‘transgene‐clean’ targeted genome‐modified soya bean plants that were homozygous for the null alleles of endogenous *GmFT2a* and without any transgenic element. These mutants of *GmFT2a* that we obtained will provide materials for more in‐depth research on *GmFT2a* functions and the molecular mechanism of photoperiod responses in soya bean. These mutants will also contribute to soya bean breeding. More ‘transgene‐clean’ mutants of desired genes could be generated using the same method, and then be cross‐fertilized to stack favourable genes. The genome‐editing machinery will significantly increase breeding efficiency and speed up breeding process.

## Results

### Targeted mutagenesis of *GmFT2a* induced by CRISPR/Cas9

**Figure 1:**
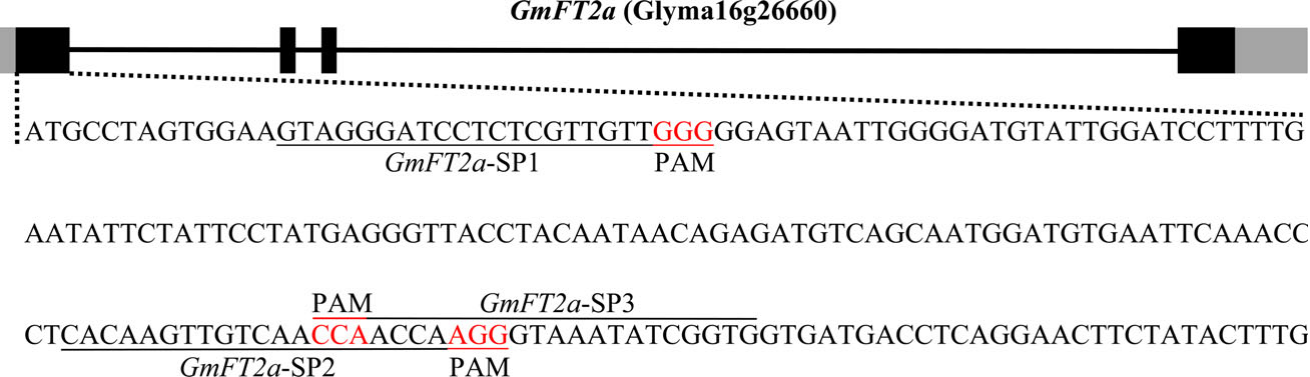
Gene structures of *GmFT2a* with target sites of CRISPR/Cas9 designed in the first exon. Black stripe, exon. Black line, intron. Grey stripe, UTR (untranslated regions). The underlined nucleotides indicate the target sites (named *GmFT2a*‐SP1, *GmFT2a*‐SP2 and *GmFT2a*‐SP3). Nucleotides in red represent PAM sequences. PAM, protospacer adjacent motif.

**Figure 2:**
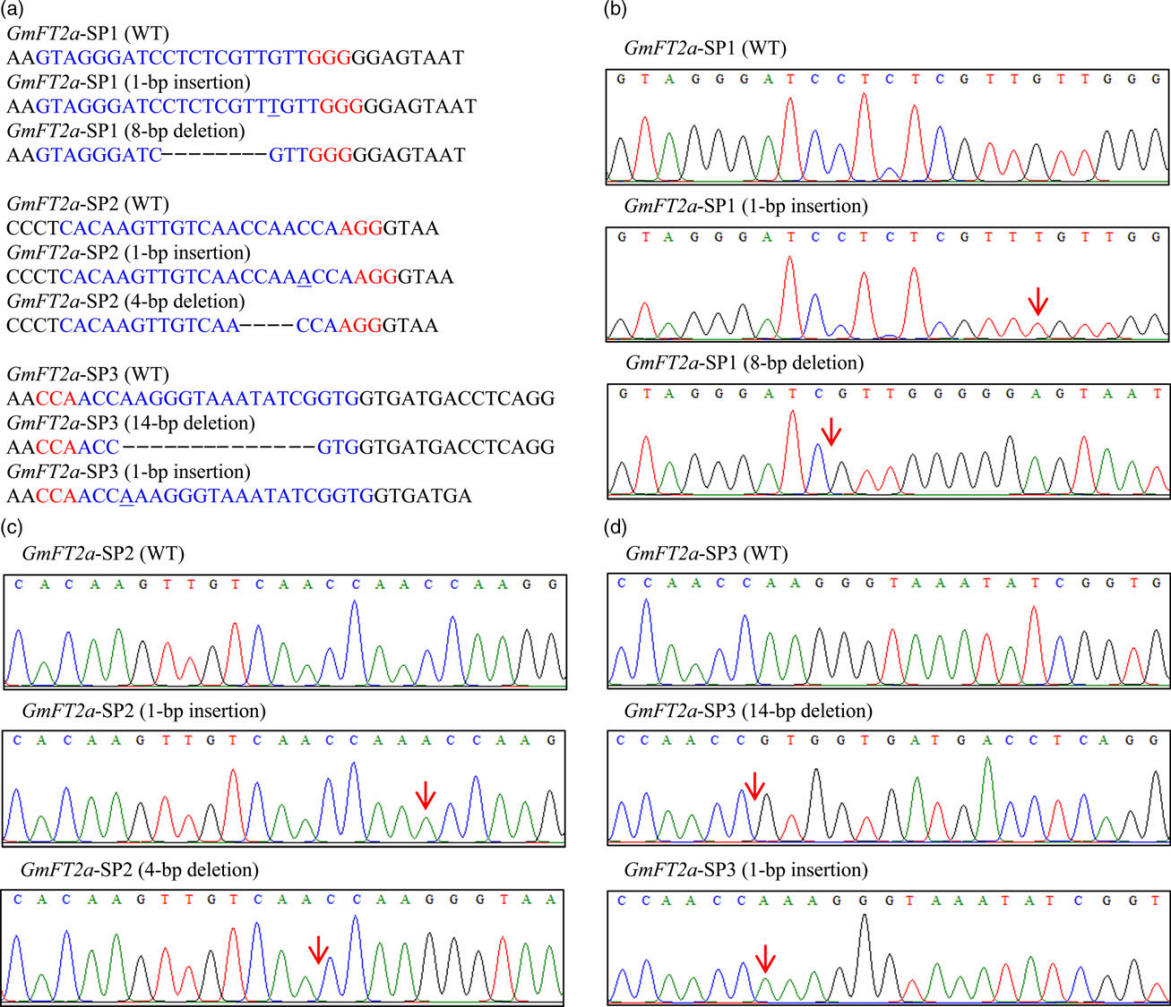
Homozygous targeted mutagenesis of *GmFT2a* induced by CRISPR/Cas9. (a) Sequences of wild type and representative mutation types induced at target sites *GmFT2a*‐SP1, *GmFT2a*‐SP2 and *GmFT2a*‐SP3 are presented, respectively. Underline, insertions. Dashes, deletions. (b), (c) and (d) are sequence peaks of wild type and representative mutation types at target sites *GmFT2a*‐SP1, *GmFT2a*‐SP2 and *GmFT2a*‐SP3, respectively. The red arrowheads indicate the location of mutations.

The CRISPR/Cas9‐mediated genome‐editing tool was utilized to knockout the soya bean endogenous gene *GmFT2a*. Three target sites (named *GmFT2a*‐SP1, *GmFT2a*‐SP2 and *GmFT2a*‐SP3) in the first exon of *GmFT2a* were chosen (Figure 1), and the corresponding sgRNA/Cas9 vectors were transformed into the soya bean cultivar Jack via *Agrobacterium tumefaciens*‐mediated transformation. In this study, the mutants of *GmFT2a* induced by CRISPR/Cas9 at the three target sites were separately named *ft2a*‐SP1, *ft2a*‐SP2 and *ft2a*‐SP3. DNA extracted from leaf tissue was used to examine CRISPR/Cas9‐induced mutations at the target sites using PCR and DNA sequencing analysis. The T0 transgenic lines harbouring the T‐DNA of the sgRNA/Cas9 vectors were identified, and we determined that 48% (12 of 25), 53% (19 of 36) and 37% (11 of 30) T‐DNA‐positive T0 lines at the three target sites had heterozygous‐targeted mutations of *GmFT2a*, respectively. Subsequently, all seeds collected from these self‐pollinated T0 lines were planted under natural conditions (summer) in Beijing, China (N39°58′, E116°20′). Site‐directed mutagenesis of *GmFT2a* was also observed at these three target sites in the T1 generation (Table 1). We identified a total of 64 T1 plants (18 *ft2a*‐SP1, 35 *ft2a*‐SP2 and 11 *ft2a*‐SP3) that were homozygous for null alleles of *GmFT2a* induced by CRISPR/Cas9 and detected two types of mutations at target site *GmFT2a*‐SP1 (8‐bp deletion and 1‐bp insertion). The 1‐bp insertion type was most frequently identified (Figure 2a,b). Simultaneously, two types of mutations were found at target site *GmFT2a*‐SP2 (4‐bp deletion and 1‐bp insertion), and the 1‐bp insertion type was most frequently identified (Figure 2a,c). Two types of mutations were also found at target site *GmFT2a*‐SP3 (14‐bp deletion and 1‐bp insertion), and the 14‐bp deletion type was most frequently identified (Figure 2a,d). All six types of frameshift mutations induced by CRISPR/Cas9 at three target sites of *GmFT2a* generated premature translation termination codons (PTCs) (Text S1).

**Table 1:** CRISPR/Cas9‐mediated targeted mutagenesis of *GmFT2a* in the T1 generation

T1 lines with *ft2a* mutations	No. of plants sequenced	No. of homozygous *ft2a* mutants	No. of heterozygous *ft2a* mutants	No. of plants with no mutation
*ft2a*‐SP1‐T1#5	17	0	11	6
*ft2a*‐SP1‐T1#10	14	2	10	2
*ft2a*‐SP1‐T1#11	53	16	25	12
*ft2a*‐SP1‐T1#16	24	0	18	6
*ft2a*‐SP1‐T1#23	8	0	8	0
*ft2a*‐SP2‐T1#8	12	8	2	2
*ft2a*‐SP2‐T1#10	11	1	8	2
*ft2a*‐SP2‐T1#13	13	0	10	3
*ft2a*‐SP2‐T1#14	5	0	1	4
*ft2a*‐SP2‐T1#15	5	3	0	2
*ft2a*‐SP2‐T1#16	12	12	0	0
*ft2a*‐SP2‐T1#22	36	10	20	6
*ft2a*‐SP2‐T1#36	27	1	9	17
*ft2a*‐SP3‐T1#2	8	0	2	6
*ft2a*‐SP3‐T1#4	6	0	3	3
*ft2a*‐SP3‐T1#10	3	0	2	1
*ft2a*‐SP3‐T1#12	8	0	5	3
*ft2a*‐SP3‐T1#30	29	11	18	0

### Potential off‐target analysis

To examine the specificity of CRISPR/Cas9 in soya bean and avoid affecting phenotype statistics by including off‐target sites, we analysed the potential off‐target effects of the editing of *GmFT2a* based on the predictions of the web tool CRISPR‐P (http://cbi.hzau.edu.cn/crispr/). The two most likely off‐target sites of these three target sites of *GmFT2a* were selected and examined by site‐specific genomic PCR and sequencing in the 64 T1 plants identified as homozygous *ft2a* mutants. All of the examined potential off‐target sites only possessed mismatches of 2–4 bp compared with the on‐target guide sequences. In this study, no mutations were observed in the examined potential off‐target sites (Table S1).

### Stable inheritance of induced mutations and phenotypes of the mutants

**Figure 3:**
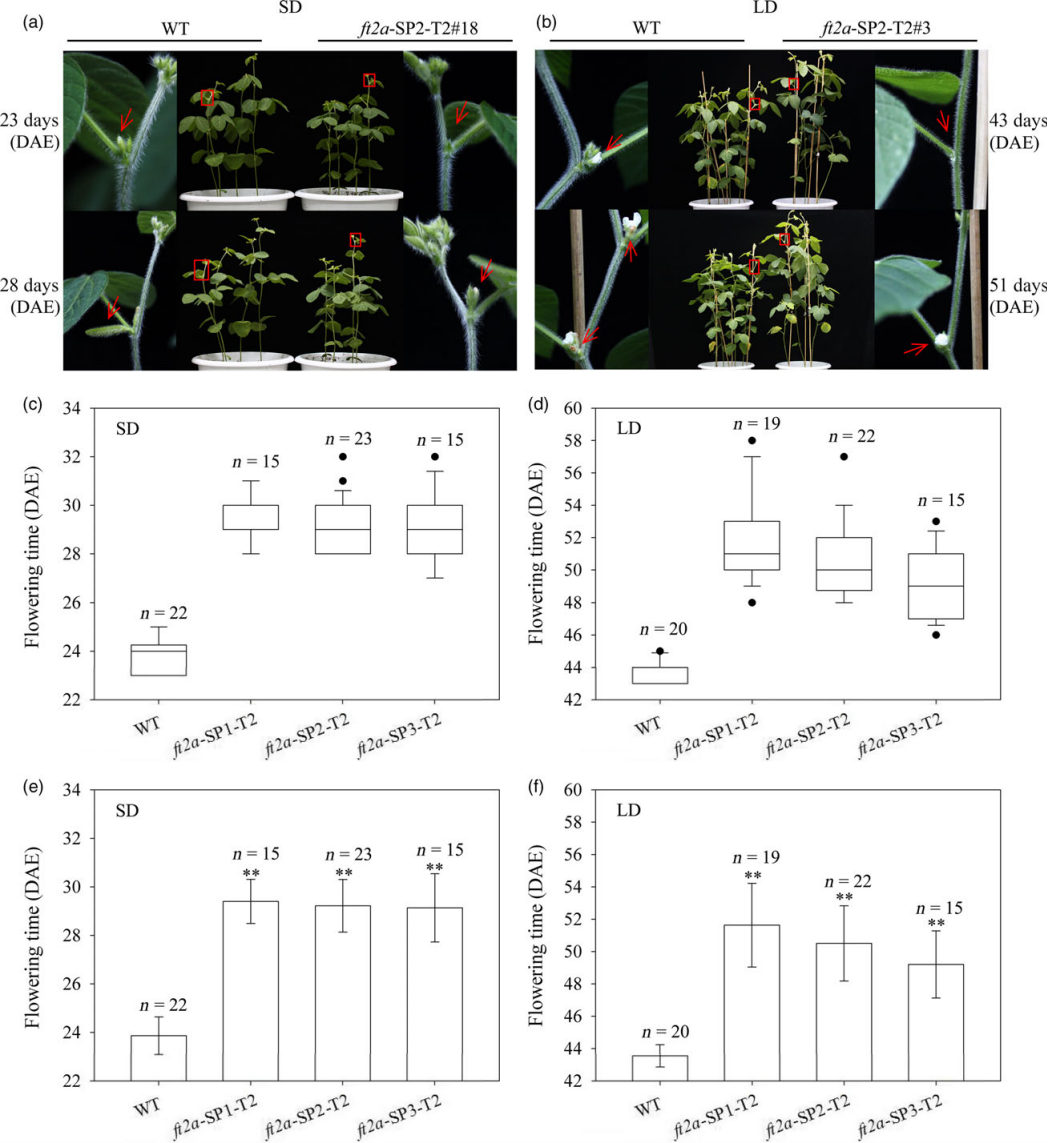
CRISPR/Cas9‐induced *ft2a* mutants exhibited late flowering in the T2 generation under both long‐day (LD) and short‐day (SD) conditions. (a) and (b) Phenotypes of wild‐type plants (WT, Jack) and homozygous T2 *ft2a*‐SP2 mutants under SD and LD condition, respectively. Top panel, *ft2a*‐SP2‐T2#18 and *ft2a*‐SP2‐T2#3 mutants did not have floral buds when WT plants were flowering. Bottom panel, flowers of WT fell off and produced the pods when *ft2a*‐SP2‐T2#18 and *ft2a*‐SP2‐T2#3 mutants were flowering. Red box, magnified view. (c) and (e) Flowering time of WT and homozygous T2 *ft2a* mutants at three target sites under SD conditions. (d) and (f) Flowering time of WT and homozygous T2 *ft2a* mutants at three target sites under LD conditions. n, exact numbers of individual plants identified. **, homozygous T2 *ft2a* mutants exhibit highly significant late flowering (*P *<* *0.01). DAE, days after emergence. The flowering time is shown as the mean values ± standard deviation.

In the T1 generation, we compared the flowering time between homozygous *ft2a* mutants induced by CRISPR/Cas9 at the three target sites with wild‐type (WT) plants under natural conditions (summer) in Beijing, China (N39°58′, E116°20′). We found that homozygous mutagenesis of *GmFT2a* at the three target sites delayed flowering time (Figure S1). To determine whether the *ft2a* mutants can transmit the induced mutations and phenotypes to their progenies, the T2 progeny of homozygous *ft2a*‐SP1‐T1#11 (1‐bp insertion), *ft2a‐SP2*‐T1#16 (1‐bp insertion) and *ft2a*‐SP3‐T1#30 (14‐bp deletion) lines were grown under both long‐day (LD, 16 h light/8 h dark) and short‐day (SD, 12 h light/12 h dark) photoperiodic conditions. The T2 plants of each mutation type were derived from three individuals of corresponding T1 *ft2a* mutant lines, respectively (34 *ft2a*‐SP1‐T2 plants were derived from *ft2a*‐SP1‐T1#11.14, *ft2a*‐SP1‐T1#11.15 and *ft2a*‐SP1‐T1#11.16 plants; 45 *ft2a*‐SP2‐T2 plants were derived from *ft2a*‐SP2‐T1#16.04, *ft2a*‐SP2‐T1#16.11 and *ft2a*‐SP2‐T1#16.12 plants; 30 *ft2a*‐SP3‐T2 plants were derived from *ft2a*‐SP3‐T1#30.02, *ft2a*‐SP3‐T1#30.08 and *ft2a*‐SP3‐T1#30.12 plants). Exact numbers of individuals are listed in Table S2. We subsequently examined the genotypes of the 109 T2‐generation plants and found that targeted mutagenesis of *GmFT2a* induced by CRISPR/Cas9 was stably inherited and maintained consistent mutation types from the T1 generation to T2 generation. Under SD conditions, T2 *ft2a* mutants did not have floral buds when WT plants were flowering, and when T2 *ft2a* mutants began to flower, the WT plants had obvious pods (Figure 3a). Similarly, under LD conditions, T2 *ft2a* mutants did not have floral buds when WT plants were flowering, and when T2 *ft2a* mutants came into flower, the flowers of WT plants fell off and began to produce pods (Figure 3b). We then compared the flowering time between T2 homozygous *ft2a* mutants at three target sites with WT plants under both SD and LD conditions via box‐plots (Figure 3c,d) and histograms (Figure 3e,f). These results exhibited that the T2 homozygous *ft2a* mutant plants showed a delayed flowering phenotype under both SD and LD conditions (Figure 3).

### Expression patterns of *GmFT2a* in WT plants and T2 homozygous *ft2a* mutants

**Figure 4:**
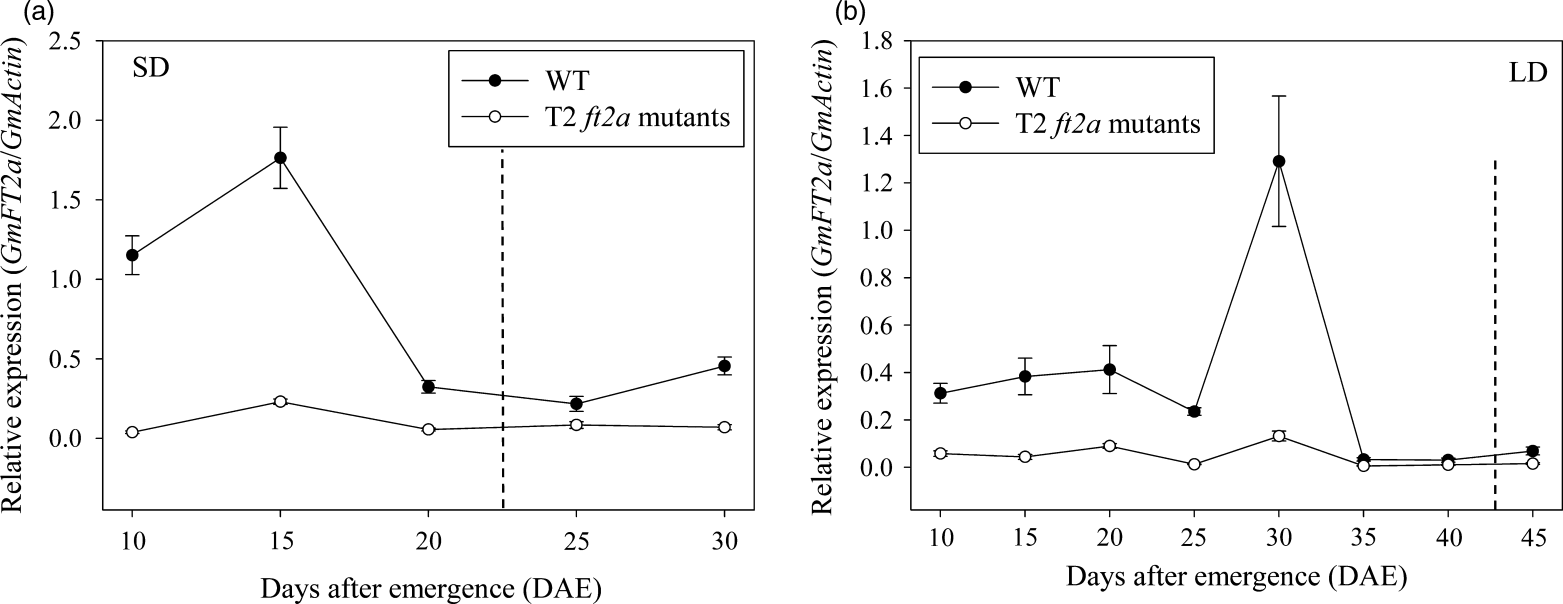
Expression patterns of *GmFT2a* in WT plants and T2 homozygous *ft2a* mutants under SD and LD conditions. (a) Expression analysis of *GmFT2a* under SD conditions. (b) Expression analysis of *GmFT2a* under LD conditions. Dotted lines indicate the flowering time of WT plants. DAE, days after emergence. RNAs were extracted from trifoliate leaves sampled at 4 h after light every 5 days beginning at 10 DAE. Relative transcript levels of *GmFT2a* were analysed by qRT‐PCR and normalized to *GmActin*. Average and SE (standard error) values for three replications are shown for each data point.

In this study, we compared the time course‐dependent expression patterns of *GmFT2a* between WT plants and T2 homozygous *ft2a* mutants grown under LD and SD photoperiodic conditions using RNAs extracted from trifoliate leaves sampled at 4 h after light every 5 days beginning at 10 DAE (Days after emergence). Under SD conditions, the expression levels of *GmFT2a* in WT plants increased to their maximum levels at 15 DAE (about 1 week before flowering) and thereafter decreased. The expression levels of *GmFT2a* in T2 *ft2a* mutants exhibited a similar pattern; however, they showed extremely low levels comparing with WT plants (Figure 4a). Under LD conditions, the expression of *GmFT2a* was relatively low. The transcript levels of *GmFT2a* in WT plants increased to their maximum levels at 30 DAE (About 2 weeks before flowering) and decreased thereafter. The transcript levels of *GmFT2a* in T2 *ft2a* mutants were significantly lower than that in WT plants, although they also increased slightly at 30 DAE (Figure 4b).

### Generation of ‘transgene‐clean’ mutant soya bean lines

**Figure 5:**
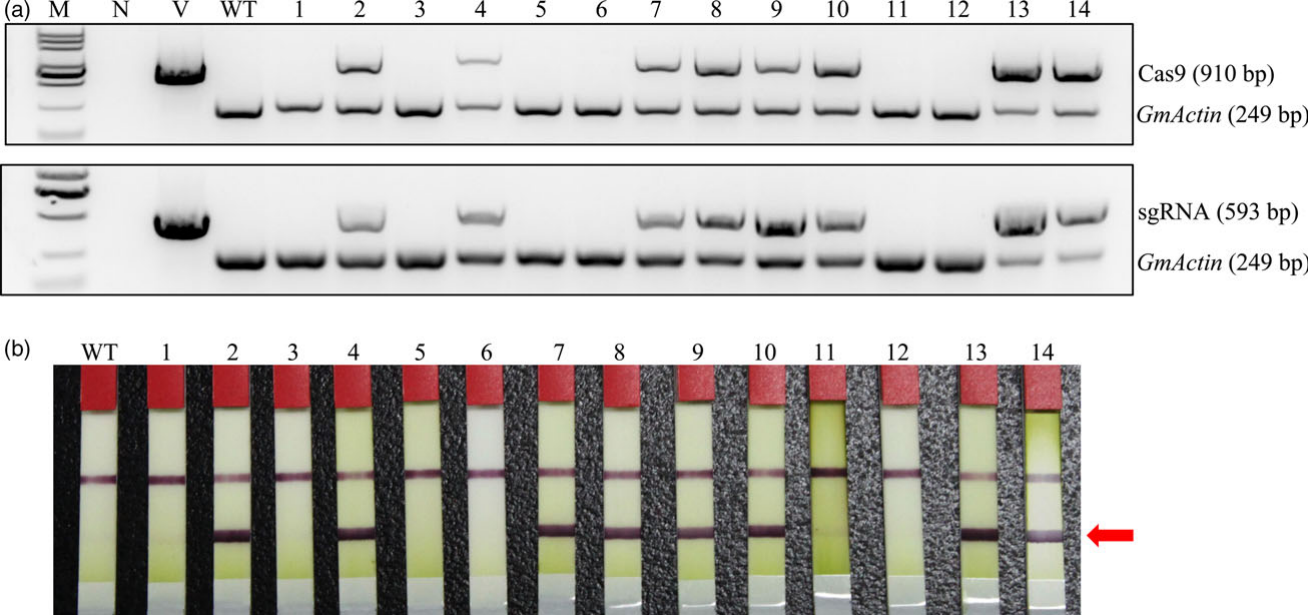
Identifying ‘transgene‐clean’ mutant soya bean lines of *GmFT2a*. (a) Gel image of PCR products obtained with primer sets for two T‐DNA regions of sgRNA/Cas9 vectors. Cas9 (910 bp), part of the Cas9 coding sequence. sgRNA (593 bp), region from the *AtU6* promoter to the downstream vector sequence spanning the sgRNA. *GmActin* was used as a normalization control. M, DL2000 ladder DNA marker. N, negative control (water as template). V, plasmid of the vector used in transformation as template. WT, DNA of wild‐type soya bean plant as template. Labels above the gel: 1–14, individual mutant lines. (b) Detection of the selectable marker gene *bar* by test strip. WT, wild‐type soya bean plants. Labels 1–14, individual mutant lines. The bands at red arrowhead indicate that *bar* is positive.

To obtain soya bean lines homozygous for endogenous *GmFT2a* mutations but without any transgenic element of the *GmFT2a*‐sgRNA/Cas9 vectors, ‘transgene‐clean’ plants were sought via a PCR strategy that used two sets of primer pairs spanning two distinct regions in the T‐DNA of sgRNA/Cas9 vectors, that is, part of the Cas9 coding sequence and region from the *AtU6* promoter to the downstream vector sequence spanning the sgRNA (Figure 5a). The selectable marker gene *bar* was examined by test strip (Figure 5b). In this assay, we found that four of 64 T1 homozygous *ft2a* mutants were ‘transgene‐clean’ and that their 46 offspring plants were all ‘transgene‐clean’ homozygous *ft2a* mutants. The 63 progeny plants of five T‐DNA‐positive T1 homozygous mutants of *GmFT2a* were also examined, and we obtained 13 ‘transgene‐clean’ homozygous *ft2a* mutants (Table 2).

**Table 2:** Identifying ‘transgene‐clean’ homozygous *ft2a* mutants from T1 and T2 generations

T1 homozygous *ft2a* mutants	T‐DNA in the T1 homozygous *ft2a* mutants	No. of the progeny plants identified	No. of the T2 ‘transgene‐clean’ homozygous *ft2a* mutants
*ft2a*‐SP1‐T1#11.14	T‐DNA‐free	7	7
*ft2a*‐SP2‐T1#16.04	T‐DNA‐free	15	15
*ft2a*‐SP3‐T1#30.08	T‐DNA‐free	12	12
*ft2a*‐SP3‐T1#30.12	T‐DNA‐free	12	12
*ft2a*‐SP1‐T1#11.15	T‐DNA‐positive	14	3
*ft2a*‐SP1‐T1#11.16	T‐DNA‐positive	13	1
*ft2a*‐SP2‐T1#16.11	T‐DNA‐positive	15	6
*ft2a*‐SP2‐T1#16.12	T‐DNA‐positive	15	2
*ft2a*‐SP3‐T1#30.02	T‐DNA‐positive	6	1

## Discussion

We previously demonstrated that CRISPR/Cas9 system shared the same efficiency for both endogenous and exogenous genes in soya bean hairy roots (Cai *et al*., 2015), but the heritability of CRISPR/Cas9‐induced mutations cannot be studied in soya bean hairy roots because they cannot regenerate. In this study, we describe a rapid and highly specific method for targeted mutagenesis of soya bean endogenous genes by CRISPR/Cas9 in whole‐plant transformation. We determined that the CRISPR/Cas9‐induced mutations were inherited from the T0 to T1 generation. However, not all T0 lines could transmit the targeted mutations to the T1 generation. Targeted mutagenesis of *GmFT2a* in only 5 of 12, 8 of 19 and 5 of 11 T0 heterozygous *ft2a* mutant lines induced by CRISPR/Cas9 at the three target sites, respectively, was transmitted to the T1 generation (Table 1). These results demonstrated that the heritability of targeted mutations generated by CRISPR/Cas9 in soya bean from the T0 to T1 generation is difficult to predict. It is possible that in the T0 generation, CRISPR/Cas9‐induced mutations exist only in a proportion of cells, which subsequently generate somatic sectors via cell division (Gaj *et al*., 2013). If somatic mutant sectors occur in the floral primordia, CRISPR/Cas9‐induced targeted mutations could be transmitted to the T1 generation through the gametes. In contrast, the targeted mutations of *GmFT2a* induced by CRISPR/Cas9 were stably inherited and maintained consistent mutation types from the T1 to T2 generation, indicating a standard germ‐line transmission pattern. Taken together, our results described a feasible strategy to introduce transmissible mutations induced by CRISPR/Cas9 using the propagation of stable transgenic plants and it may be widely used for reverse genetics in soya bean.

Off‐target events are notable in the application of CRISPR/Cas9. To examine the specificity of CRISPR/Cas9 in soya bean and avoid affecting phenotype statistics by including off‐target sites, we analysed the potential off‐target effects of the editing of *GmFT2a*. The two most likely off‐target sites of the three target sites of *GmFT2a* were respectively selected and examined. We observed no mutations at all of the putative off‐target sites. Previous reports have demonstrated that the length of the sgRNA and the homology between the sgRNA and candidate off‐target site are the main influencing factors (Zhang *et al*., 2014). A double Cas9 nickase approach has also been reported to effectively reduce off‐target effects (Fauser *et al*., 2014). Whole‐genome sequencing should be performed if off‐target effects are fatal, especially in clinical research. However, it may not be a fatal problem in plant basic research. The risk of off‐target events induced by CRISPR/Cas9 in plants may not be high on account of the lower somatic mutation frequency in tissue culture‐based transformation or other mutagenic treatments (Ma *et al*., 2015). Through careful target selection, potential off‐target mutations could be minimized (Xie *et al*., 2014; Xu *et al*., 2015). In addition, undesired off‐target mutations in plants could be eliminated by hybridization, and off‐target events could be utilized to knockout multiple genes simultaneously. In previous studies, we demonstrated the potential of CRISPR/Cas9 to simultaneously edit two endogenous soya bean genes using only one customized sgRNA based on the off‐target principle (Cai *et al*., 2015). In short, the adverse effects of off‐target events could be eliminated in plant basic research.

In previous studies, site‐directed mutagenesis of *FLOWER LOCUS T* (*FT*) generated by CRISPR/Cas9 delayed flowering time in *A. thaliana* (Hyun *et al*., 2015). *FT* encodes florigen and plays an important role in flowering pathways as an integrator (Corbesier and Coupland, 2006; Turck *et al*., 2008). The FT protein is a florigen that moves through the phloem to the shoot apex and works as a long‐distance signal to induce floral initiation (Corbesier *et al*., 2007; Jaeger and Wigge, 2007; Mathieu *et al*., 2007; Notaguchi *et al*., 2008). *GmFT2a* is a soya bean ortholog of *FT* and plays an important role in flowering induction in soya bean (Kong *et al*., 2010; Nan *et al*., 2014; Sun *et al*., 2011). In the present study, three target sites in the first exon of *GmFT2a* were chosen. These mutations induced by CRISPR/Cas9 in the coding region were expected to inactivate the GmFT2a protein function by inducing frameshift mutations. We compared the flowering time between homozygous *ft2a* mutants with WT plants and found that the homozygous *ft2a* mutants exhibited late‐flowering phenotype under natural, SD and LD conditions. We also compared the time course‐dependent expression patterns of *GmFT2a* between WT plants and T2 homozygous *ft2a* mutants grown under LD and SD photoperiodic conditions using RNAs extracted from trifoliate leaves sampled at 4 h after light every 5 days beginning at 10 DAE. The results showed that the expression levels of *GmFT2a* increased to their maximum levels and thereafter decreased before flowering under both LD and SD photoperiodic conditions. What's more, the transcript levels of *GmFT2a* in T2 *ft2a* mutants were significantly lower than that in WT plants. In eukaryotic cells, non‐sense‐mediated mRNA decay (NMD) is an evolutionarily well‐conserved and effective mRNA surveillance pathway that ensures the detection and rapid degradation of mRNAs containing premature translation termination codons (PTCs), thereby preventing the potentially deleterious effects of truncated proteins (Conti and Izaurralde, 2005; Maquat, 2005). In this study, all six types of frameshift mutations induced by CRISPR/Cas9 at three target sites of *GmFT2a* generated PTCs (Text S1). Therefore, we predicted that the mRNA of *GmFT2a* containing PTCs was degraded by the NMD surveillance mechanism and then showed extremely low expression levels. *GmFT2a* likely encodes florigen, and thus loss of *GmFT2a* gene function resulted in a late‐flowering phenotype.

We obtained some ‘transgene‐clean’ targeted genome‐modified soya bean plants that were homozygous for the null alleles of endogenous *GmFT2a* and without any transgenic element in the T1 and T2 generations using the CRISPR/Cas9 system. In recent years, transgenic technology has provided many important technical breakthroughs and progress to overcome the shortcomings of traditional breeding methods, such as long cycle, labour intensive and low efficiency. However, there are also some disadvantages to this technology. Foreign genes are typically randomly integrated into the plant genome after entering the nucleus, which may generate negative results, such as the disruption of plant endogenous genes or exogenous gene silencing (Napoli *et al*., 1990). Although there is no scientific evidence that genetically modified organism (GMO) products are harmful to human health, debates about GMO safety continue. Genome‐editing techniques, especially CRISPR/Cas9, provide a new approach to solve this problem. The CRISPR/Cas9 system could accurately generate site‐directed mutations in specific genes and exogenous elements such as sgRNA. Cas9 and the associated selectable marker can be removed in later generations via genetic segregation by a selfing or hybridization approach. The ‘transgene‐clean’ mutants have been generated by the CRISPR/Cas9 system in many plant species, such as *A. thaliana* (Gao *et al*., 2016; Pyott *et al*., 2016), rice (Li *et al*., 2016; Xu *et al*., 2015; Zhou *et al*., 2016), wheat (Zhang *et al*., 2016) and tomato (Soyk *et al*., 2017). The ‘transgene‐clean’ soya bean varieties with high oleic acid have been created by targeted mutagenesis with transcription activator‐like effector nucleases (TALENs) (Haun *et al*., 2014). Similarly, CRISPR/Cas9 could also be utilized to improve soya bean varieties, such as quality optimization, disease resistance and growth period traits. In the present study, we obtained ‘transgene‐clean’ soya bean plants which were delayed flowering time by homozygous targeted mutagenesis of endogenous *GmFT2a* with CRISPR/Cas9. These mutants also provide materials for further in‐depth research; for example, they can be utilized to explore the movement of *GmFT2a* by importing the full *GmFT2a* expression cassette in combination with GFP (green fluorescent protein) and studying functional complementation. The same method could be used to generate more ‘transgene‐clean’ mutants of *GmFT2a*‐relevant genes by CRISPR/Cas9, which could then be crossed to research gene function. Soya bean is very sensitive to photoperiod, which limits its geographical range of cultivation, and our studies may contribute to soya bean regional introduction and domestication.

## Experimental procedures

### Plant materials and growth conditions

The soya bean cultivar Jack was used for transformation in this study. Wild‐type Jack (as a control) and all seeds collected from *ft2a*‐CRISPR/Cas9 T0 plants were sown under natural conditions in Beijing, China (N39°58′, E116°20′) on 11 May 2016. The progeny of T1 homozygous *ft2a* mutants was separately grown under long‐day (16 h light/8 h dark) and short‐day (12 h light/12 h dark) photoperiodic conditions at 27 °C with 50% relative humidity.

### SgRNA design and construction of the CRISPR/Cas9 expression vector

To construct a plasmid vector carrying both sgRNA and Cas9 cassettes, the sequence of Cas9 was codon‐optimized for dicotyledons and assembled downstream of the CaMV 2× 35S promoter together with a customized sgRNA driven by the *Arabidopsis U6* promoter (Text S2). The *bar* gene driven by a CaMV 35S promoter was used as a screening marker. The schematic of *GmFT2a*‐CRISPR/Cas9 vector is shown in Figure S2. The sequences were synthesized by ViewSolid Biotech (Beijing). The sequence and other information for the analysed soya bean endogenous gene *GmFT2a* were downloaded from the Phytozome website (www.phytozome.net/). We designed these sgRNAs using the web tool CRISPR‐P (http://cbi.hzau.edu.cn/crispr/) (Lei *et al*., 2014), which displayed all optional sgRNA sequences (20 bp) immediately followed by 5′‐NGG (PAM, protospacer adjacent motif) in the forward or reverse strand. In this study, we selected three sgRNAs targeting *GmFT2a*, named as *GmFT2a*‐SP1, *GmFT2a*‐SP2 and *GmFT2a*‐SP3. The first base was a guanine (G) nucleotide in *GmFT2a*‐SP1 but not *GmFT2a*‐SP2 and *GmFT2a*‐SP3. Thus, an extra G was appended before the 5′ end of the sequences of these two sgRNAs (Ran *et al*., 2013). For each sgRNA, a pair of DNA oligonucleotides was synthesized by the Beijing Genomics Institute (BGI) and annealed to generate dimers, which were subsequently integrated upstream of the sgRNA scaffolds in the plasmid vector simultaneously expressing Cas9 and sgRNA. After transformation into *E. coli* DH5α for *in vivo* cloning, these CRISPR/Cas9 expression vectors were purified using the EasyPure Plasmid MiniPrep Kit (TransGen Biotech) for subsequent use in soya bean transformation.

### Transformation of CRISPR/Cas9 in soya bean and screening for mutations by sequencing analysis

CRISPR/Cas9 expression vectors were individually transformed into *Agrobacterium tumefaciens* strain EHA105 via electroporation. The soya bean cultivar Jack was used for tissue culture and transformation according to the protocol previously reported (Song *et al*., 2013). Genomic DNA was extracted from the leaves of each individual plant in the T0 generation, and then the regions spanning the target sites were amplified by PCR using Phanta^®^ Super Fidelity DNA Polymerase (Vazyme Biotech) with the *GmFT2a* forward primer (5′‐ATTCATAACAAAGCAAACGAG‐3′) and reverse primer (5′‐ACTTGACCTTCCCTTAAACAC‐3′), purified and sequenced. Different types of gene editing can be identified via sequence peaks. Short base insertions or deletions (not multiples of three) induced by CRISPR/Cas9 can lead to frameshift mutations. The heterozygous mutations showed overlapping peaks from the target sites to the end. The wild‐type and homozygous mutations had no overlapping peaks at the target sites. Then, the homozygous mutant types were identified by sequence alignment with the wild‐type sequence. This method was also used in the T1 and T2 generations.

### Flowering time measurements and statistical analyses

The flowering time of each soya bean plant was recorded as days from emergence to the R1 stage (the first flower appears at any node in the main stem; Fehr *et al*., 1971). For quantitative analyses of flowering time, at least 11 individual soya bean plants were analysed per genotype, and exact numbers of individuals (n) are shown in Figure 3 and Figure S1. Statistical analyses were performed using Microsoft Excel. A one‐way analysis of variance least significant difference test (LSD) was used to compare the significance of differences between controls and treatments at the 0.01 probability level. SigmaPlot 10.0 was used for drawing box‐plots and histograms. The flowering time is shown as the mean values ± standard deviation.

### Gene expression analysis by quantitative real‐time PCR (qRT‐PCR)

The wild‐type plants and T2 homozygous *ft2a* mutants grown under long‐day (16 h light/8 h dark) and short‐day (12 h light/12 h dark) photoperiodic conditions were used to compare the expression levels of *GmFT2a*. Pieces of fully developed trifoliate leaves at the upper part of 16 wild‐type plants (Every 8 individual plants grown under LD and SD conditions, respectively) and 24 T2 *ft2a* mutants (Every 12 individual plants grown under LD and SD conditions, respectively) were sampled at 4 h after light every 5 days beginning at 10 DAE. These leaves were immediately frozen in liquid nitrogen. Total RNA was isolated from frozen tissue using TransZol Up Plus RNA Kit (TransGen Biotech). For reverse transcription, 1 μg of total RNA was used to synthesize single‐stranded cDNA using TransScript One‐Step gDNA Removal and cDNA Synthesis SuperMix (TransGen Biotech). For qRT‐PCR, each 10‐μL reaction contained 1 μL of 1 : 5 diluted cDNA with 0.2 μL of each primer (10 μm), 5 μL of 2× KAPA SYBR^®^ FAST qPCR Master Mix, 0.2 μL of 50× ROX High Reference Dye (KAPA Biosystems), and water to a final volume of 10 μL. The ABI Prism 7900HT Real‐Time PCR System was used. The PCR cycling conditions started with a denaturing step for 3 min at 95 °C followed by 40 cycles of 5 s at 95 °C and a primer extension reaction at 60 °C for 30 s. All PCR reactions were run with three biological replicates each. Data were analysed using the 2^−ΔΔCt^ method with the mRNA level of *GmActin* (Glyma18g52780) gene as an internal control.

### Primer sequences used in the present study

The primer sequences used for amplifying the regions which span the target sites, potential off‐target analysis, identifying ‘transgene‐clean’ mutant soya bean lines and qRT‐PCR analysis are listed in Table S3.

## Conflict of interest

The authors declare no conflict of interest.

## Supporting information


**Figure S1** Homozygous *ft2a* mutants at three target sites in the T1 generation delayed flowering time under natural conditions (summer) in Beijing, China (N39°58′, E116°20′).
**Figure S2** Schematic illustrating the basic architecture of the constructs used for CRISPR/Cas9‐mediated genome editing.
**Table S1** Potential off‐target analysis at the three target sites of *GmFT2a* in the T1 generation.
**Table S2** T2 *ft2a* mutants under LD and SD conditions.
**Table S3** Primer sequences used in the present study.
**Text S1** Frameshift mutations at three target sites of *GmFT2a* generated premature translation termination codons (PTCs). CDS, coding sequence. Blue capital letter, target sequence. Red capital letter, protospacer adjacent motif. Underline, insertions. Dashes, deletions. Yellow rectangle, termination codon.
**Text S2** The sequences of the Cas9 and *Arabidopsis U6* promoter used in the present study.
