# Supplementary material for: CRISPR/Cas9‐mediated targeted mutagenesis of GmFT2a delays flowering time in soya bean
Source: Plant Biotechnol J. 2017 Jun 20;16(1):176–85. doi: 10.1111/pbi.12758 (PMC5785355; doi:10.1111/pbi.12758)
Supplement: Supplementary file 1 — Figure S1 Homozygous ft2a mutants at three target sites in the T1 generation delayed flowering time under natural conditions (summer) in Beijing, China (N39°58′, E116°20′). Figure S2 Schematic illustrating the basic architecture of the constructs used for CRISPR/Cas9‐mediated genome editing. Table S1 Potential off‐target analysis at the three target sites of GmFT2a in the T1 generation. Table S2 T2 ft2a mutants under LD and SD conditions. Table S3 Primer sequences used in the present study. Text S1 Frameshift mutations at three target sites of GmFT2a generated premature translation termination codons (PTCs). CDS, coding sequence. Blue capital letter, target sequence. Red capital letter, protospacer adjacent motif. Underline, insertions. Dashes, deletions. Yellow rectangle, termination codon. Text S2 The sequences of the Cas9 and Arabidopsis U6 promoter used in the present study. [file PBI-16-176-s001.pdf]

Table S1 Potential off-target analysis at the three target sites of *GmFT2a* in the T1 generation

Target site	Putative off-target site				No. of plants sequenced[c]	No. of plants with mutations
	Gene locus	Region	Sequence[a]	MMs[b]		
GmFT2a-SP1	Glyma19g24640<br>19:-30300772	intron	GTgGtGAaCCTCTCGTTGTT<br>GGG	3	18	0
	Glyma16g26690<br>16:+30780526	exon	GTAGGGAcCCTCTaGTTGT<br>TGGG	2	18	0
GmFT2a-SP2	Glyma12g35200<br>12:-38349846	utr	CACAcGTgGgaAACCAACC<br>AAGG	4	35	0
	Glyma15g10400<br>15:+7538434	intron	CtCAtGTTtTCAACCAAgCA<br>AGG	4	35	0
GmFT2a-SP3	Glyma09g11720<br>9:-11946423	utr	gtCtGATATTaACCCTTGGTT<br>GG	4	11	0
	Glyma16g26690<br>16:-30780651	exon	CACCGATATTTAttCTTGGT<br>TGG	2	11	0

[a] Mismatched bases are shown in lowercase letters. [b] No. of mismatched bases. [c] T1 plants identified as biallelic mutants of *GmFT2a*.

Text S1 Frameshift mutations at three target sites of *GmFT2a* generated premature translation termination codons (PTCs)

CDS of *GmFT2a* (WT, wild-type)

ATGCCTAGTGGAAGTAGGGATCCTCTCGTTGTTGGGGGAGTAATTGGGGATGTATTGGATCCTTTTGAATATTCTATTCC
TATGAGGGTTACCTACAATAACAGAGATGTCAGCAATGGATGTGAATTCAAACCCTCACAAGTTGTCAACCAACCAAG
GGTAAATATCGGTGGTGATGACCTCAGGAACTTCTATACTTTGATTGCGGTTGATCCCGATGCACCTAGCCCAAGTGAC
CCCAATTTGAGAGAATACCTCCATTGGTTGGTGACTGATATCCCAGCAACAACAGGGGCTAGTTTCGGCCATGAGGTT
GTAACATATGAAAGTCCAAGACCAATGATGGGGATTCATCGTTTGGTGTTTGTGTTATTTCGTCAACTGGGTAGGGAGA
CCGTGTATGCACCAGGATGGCGCCAGAATTTCAACACTAAAGAATTTGCTGAACTTTACAACCTTGGATTGCCAGTTG
CTGCTGTCTATTTCAACATTCAGAGGGAATCTGGTTCTGGTGGAAGGAGGTTATACTAA

Protein sequence of *GmFT2a* (WT, wild-type)

MPSGSRDPLVVGGVIGDVLDPFEYSIPMRVTYNNRDVSNGCEFKPSQVVNQPRVNIGGDDLRNFYTLIAVDPDAPSPSDPN
LREYLHWLVTDIPATTGASFGHEVVTYESPRPMMGIHRLVFVLFRQLGRETVYAPGWRQNFNTKEFAELYNLGLPVAAVYF
NIQRESGSGGRRLY

***GmFT2a*-SP1 (1-bp insertion)**

CDS

ATGCCTAGTGGAAGTAGGGATCCTCTCGTTTGTTGGGGGAGTAATTGGGGATGTATTGGATCCTTTTGAATATTCTATTC
CTATGAGGGTTACCTACAATAACAGAGATGTCAGCAATGGATGTGAATTCAAACCCTCACAAGTTGTCAACCAACCAA
GGGTAAATATCGGTGGTGATGACCTCAGGAACTTCTATACTTTGATTGCGGTTGATCCCGATGCACCTAGCCCAAGTGA
CCCCAATTTGAGAGAATACCTCCATTGGTTGGTGACTGATATCCCAGCAACAACAGGGGCTAGTTTCGGCCATGAGGT
TGTAACATATGAAAGTCCAAGACCAATGATGGGGATTCATCGTTTGGTGTTTGTGTTATTTCGTCAACTGGGTAGGGAG
ACCGTGTATGCACCAGGATGGCGCCAGAATTTCAACACTAAAGAATTTGCTGAACTTTACAACCTTGGATTGCCAGTT
GCTGCTGTCTATTTCAACATTCAGAGGGAATCTGGTTCTGGTGGAAGGAGGTTATACTAA

Protein sequence

MPSGSRDPLVCWGSNWGCIGSF **Stop**

***GmFT2a*-SP1 (8-bp deletion)**

CDS

ATGCCTAGTGGAAGTAGGGATC--------GTTGGGGGAGTAATTGGGGATGTATTGGATCCTTTTGAATATTCTATTCCTATG
AGGGTTACCTACAATAACAGAGATGTCAGCAATGGATGTGAATTCAAACCCTCACAAGTTGTCAACCAACCAAGGGTA
AATATCGGTGGTGATGACCTCAGGAACTTCTATACTTTGATTGCGGTTGATCCCGATGCACCTAGCCCAAGTGACCCCA
ATTTGAGAGAATACCTCCATTGGTTGGTGACTGATATCCCAGCAACAACAGGGGCTAGTTTCGGCCATGAGGTTGTAA
CATATGAAAGTCCAAGACCAATGATGGGGATTCATCGTTTGGTGTTTGTGTTATTTCGTCAACTGGGTAGGGAGACCGT
GTATGCACCAGGATGGCGCCAGAATTTCAACACTAAAGAATTTGCTGAACTTTACAACCTTGGATTGCCAGTTGCTGC
TGTCTATTTCAACATTCAGAGGGAATCTGGTTCTGGTGGAAGGAGGTTATACTAA

Protein sequence

MPSGSRDRWGSNWGCIGSF **Stop**

***GmFT2a*-SP2 (1-bp insertion)**

CDS

ATGCCTAGTGGAAGTAGGGATCCTCTCGTTGTTGGGGGAGTAATTGGGGATGTATTGGATCCTTTTGAATATTCTATTCC
TATGAGGGTTACCTACAATAACAGAGATGTCAGCAATGGATGTGAATTCAAACCCTCACAAGTTGTCAACCAAACCAA
GGGTAAATATCGGTGGTGATGACCTCAGGAACTTCTATACTTTGATTGCGGTTGATCCCGATGCACCTAGCCCAAGTGA
CCCCAATTTGAGAGAATACCTCCATTGGTTGGTGACTGATATCCCAGCAACAACAGGGGCTAGTTTCGGCCATGAGGT
TGTAACATATGAAAGTCCAAGACCAATGATGGGGATTCATCGTTTGGTGTTTGTGTTATTTCGTCAACTGGGTAGGGAG

ACCGTGTATGCACCAGGATGGCGCCAGAATTTCAACACTAAAGAATTTGCTGAACTTTACAACCTTGGATTGCCAGTT
GCTGCTGTCTATTTCAACATTCAGAGGGAATCTGGTTCTGGTGGAAGGAGGTTATACTAA

Protein sequence

MPSGSRDPLVVGGVIGDVLDPFEYSIPMRVTYNNRDVSNGCEFKPSQVVNQTKGKYRW **Stop**

***GmFT2a*-SP2 (4-bp deletion)**

CDS

ATGCCTAGTGGAAGTAGGGATCCTCTCGTTGTTGGGGGAGTAATTGGGGATGTATTGGATCCTTTTGAATATTCTATTCC
TATGAGGGTTACCTACAATAACAGAGATGTCAGCAATGGATGTGAATTCAAACCCTCACAAGTTGTCAA----CCAAGGG
TAAATATCGGTGGTGATGACCTCAGGAACTTCTATACTTTGATTGCGGTTGATCCCGATGCACCTAGCCCAAGTGACCC
CAATTTGAGAGAATACCTCCATTGGTTGGTGACTGATATCCCAGCAACAACAGGGGCTAGTTTCGGCCATGAGGTTGT
AACATATGAAAGTCCAAGACCAATGATGGGGATTCATCGTTTGGTGTTTGTGTTATTTCGTCAACTGGGTAGGGAGACC
GTGTATGCACCAGGATGGCGCCAGAATTTCAACACTAAAGAATTTGCTGAACTTTACAACCTTGGATTGCCAGTTGCT
GCTGTCTATTTCAACATTCAGAGGGAATCTGGTTCTGGTGGAAGGAGGTTATACTAA

Protein sequence

MPSGSRDPLVVGGVIGDVLDPFEYSIPMRVTYNNRDVSNGCEFKPSQVVNQG **Stop**

***GmFT2a*-SP3 (14-bp deletion)**

CDS

ATGCCTAGTGGAAGTAGGGATCCTCTCGTTGTTGGGGGAGTAATTGGGGATGTATTGGATCCTTTTGAATATTCTATTCC
TATGAGGGTTACCTACAATAACAGAGATGTCAGCAATGGATGTGAATTCAAACCCTCACAAGTTGTCAACCAACC-------
-------GTGGTGATGACCTCAGGAACTTCTATACTTTGATTGCGGTTGATCCCGATGCACCTAGCCCAAGTGACCCCAATTT
GAGAGAATACCTCCATTGGTTGGTGACTGATATCCCAGCAACAACAGGGGCTAGTTTCGGCCATGAGGTTGTAACATAT
GAAAGTCCAAGACCAATGATGGGGATTCATCGTTTGGTGTTTGTGTTATTTCGTCAACTGGGTAGGGAGACCGTGTATG
CACCAGGATGGCGCCAGAATTTCAACACTAAAGAATTTGCTGAACTTTACAACCTTGGATTGCCAGTTGCTGCTGTCTA
TTTCAACATTCAGAGGGAATCTGGTTCTGGTGGAAGGAGGTTATACTAA

Protein sequence

MPSGSRDPLVVGGVIGDVLDPFEYSIPMRVTYNNRDVSNGCEFKPSQVVNQPW **Stop**

***GmFT2a*-SP3 (1-bp insertion)**

CDS

ATGCCTAGTGGAAGTAGGGATCCTCTCGTTGTTGGGGGAGTAATTGGGGATGTATTGGATCCTTTTGAATATTCTATTCC
TATGAGGGTTACCTACAATAACAGAGATGTCAGCAATGGATGTGAATTCAAACCCTCACAAGTTGTCAACCAACCAAA
GGGTAAATATCGGTGGTGATGACCTCAGGAACTTCTATACTTTGATTGCGGTTGATCCCGATGCACCTAGCCCAAGTGA
CCCCAATTTGAGAGAATACCTCCATTGGTTGGTGACTGATATCCCAGCAACAACAGGGGCTAGTTTCGGCCATGAGGT
TGTAACATATGAAAGTCCAAGACCAATGATGGGGATTCATCGTTTGGTGTTTGTGTTATTTCGTCAACTGGGTAGGGAG
ACCGTGTATGCACCAGGATGGCGCCAGAATTTCAACACTAAAGAATTTGCTGAACTTTACAACCTTGGATTGCCAGTT
GCTGCTGTCTATTTCAACATTCAGAGGGAATCTGGTTCTGGTGGAAGGAGGTTATACTAA

Protein sequence

MPSGSRDPLVVGGVIGDVLDPFEYSIPMRVTYNNRDVSNGCEFKPSQVVNQPKGKYRW **Stop**

Text S1 Frameshift mutations at three target sites of *GmFT2a* generated premature translation termination codons (PTCs). CDS, coding sequence. Blue capital letter, target sequence. Red capital letter, protospacer adjacent motif. Underline, insertions. Dashes, deletions. Yellow rectangle, termination codon.

(a) (b)

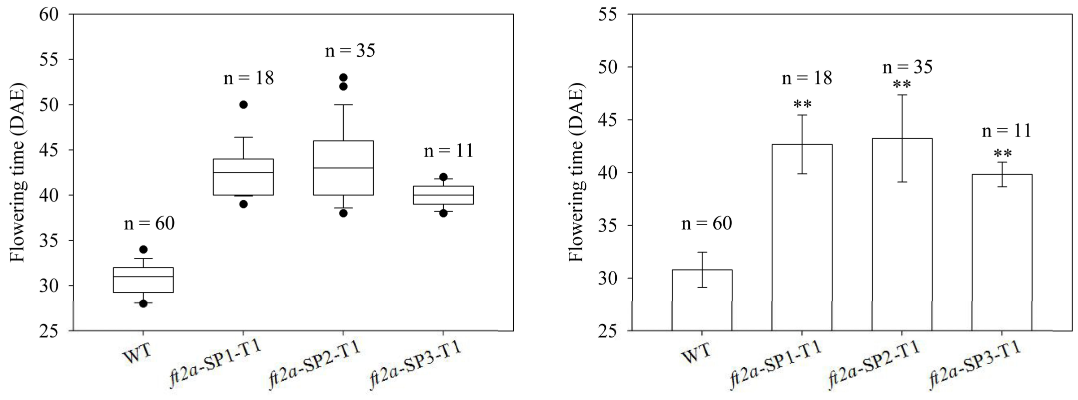

Figure S1 Homozygous *ft2a* mutants at three target sites in the T1 generation delayed flowering time under natural conditions (summer) in Beijing, China (N39°58', E116°20'). WT, wild-type plant (Jack). *ft2a*-SP1-T1, *ft2a*-SP2-T1 and *ft2a*-SP3-T1 were homozygous mutants of *GmFT2a* at the three target sites in the T1 generation, respectively. n, exact numbers of individual plants identified. The flowering time are shown as the mean values ± standard deviation. **, homozygous T1 *ft2a* mutants exhibit highly significant late flowering ($P < 0.01$). DAE, days after emergence.

Table S2 T2 *ft2a* mutants under LD and SD conditions

T1 homozygous *ft2a* mutants	The types of homozygous mutations	No. of the progeny plants under LD conditions	No. of the progeny plants under SD conditions	Total
ft2a-SP1-T1#11.14	1-bp insertion at *GmFT2a*-SP1	4	3	7
ft2a-SP1-T1#11.15	1-bp insertion at *GmFT2a*-SP1	8	6	14
ft2a-SP1-T1#11.16	1-bp insertion at *GmFT2a*-SP1	7	6	13
ft2a-SP2-T1#16.04	1-bp insertion at *GmFT2a*-SP2	7	8	15
ft2a-SP2-T1#16.11	1-bp insertion at *GmFT2a*-SP2	7	8	15
ft2a-SP2-T1#16.12	1-bp insertion at *GmFT2a*-SP2	8	7	15
ft2a-SP3-T1#30.02	14-bp deletion at *GmFT2a*-SP3	3	3	6
ft2a-SP3-T1#30.08	14-bp deletion at *GmFT2a*-SP3	6	6	12
ft2a-SP3-T1#30.12	14-bp deletion at *GmFT2a*-SP3	6	6	12
Total		56	53	109

Text S2 The sequences of the Cas9 and *Arabidopsis U6* promoter used in the present study.

Cas9 DNA sequence

atggcccctaagaagaagagaaaggtcggtattcacggcgttcctgcggcgatggacaagaagtatagtattggtctggacattgggacgaattccgttggctgggccgtgatcaccgatgagt

acaaggtcccttccaagaagtttaaggttctggggaacaccgatcggcacagcatcaagaagaatctcattggagccctcctgttcgactcaggcgagaccgccgaagcaacaaggctcaag

agaaccgcaaggagacggtatacaagaaggaagaataggatctgctacctgcaggagattttcagcaacgaaatggcgaaggtggacgattcgttctttcatagattggaggagagtttcctcg

tcgaggaagataagaagcacgagaggcatcctatctttggcaacattgtcgacgaggttgcctatcacgaaaagtaccccacaatctatcatctgcggaagaagcttgtggactcgactgataag

gcggaccttagattgatctacctcgctctggcacacatgattaagttcaggggccattttctgatcgagggggatcttaacccggacaatagcgatgtggacaagttgttcatccagctcgtccaaa

cctacaatcagctctttgaggaaaacccaattaatgcttcaggcgtcgacgccaaggcgatcctgtctgcacgcctttcaaagtctcgccggcttgagaacttgatcgctcaactcccgggcgaa

aagaagaacggcttgttcgggaatctcattgcactttcgttggggctcacaccaaacttcaagagtaattttgatctcgctgaggacgcaaagctgcagctttccaaggacacttatgacgatgacc

tggataaccttttggcccaaatcggcgatcagtacgcggacttgttcctcgccgcgaagaatttgtcggacgcgatcctcctgagtgatattctccgcgtgaacaccgagattacaaaggccccg

ctctcggcgagtatgatcaagcgctatgacgagcaccatcaggatctgacccttttgaaggctttggtccggcagcaactcccagagaagtacaaggaaatcttctttgatcaatccaagaacgg

ctacgctggttatattgacggcggggcatcgcaggaggaattctacaagtttatcaagccaattctggagaagatggatggcacagaggaactcctggtgaagctcaatagggaggaccttttg

cggaagcaaagaactttcgataacggcagcatccctcaccagattcatctcggggagctgcacgccatcctgagaaggcaggaagacttctacccctttcttaaggataaccgggagaagatc

gaaaagattctgacgttcagaattccgtactatgtcggaccactcgcccggggtaattccagatttgcgtggatgaccagaaagagcgaggaaaccatcacaccttggaacttcgaggaagtgg

tcgataagggcgcttccgcacagagcttcattgagcgcatgacaaattttgacaagaacctgcctaatgagaaggtccttcccaagcattccctcctgtacgagtatttcactgtttataacgaactc

acgaaggtgaagtatgtgaccgagggaatgcgcaagcccgccttcctgagcggcgagcaaaagaaggcgatcgtggaccttttgtttaagaccaatcggaaggtcacagttaagcagctcaa

ggaggactacttcaagaagattgaatgcttcgattccgttgagatcagcggcgtggaagacaggtttaacgcgtcactggggacttaccacgatctcctgaagatcattaaggataaggacttctt

ggacaacgaggaaaatgaggatatcctcgaagacattgtcctgactcttacgttgtttgaggatagggaaatgatcgaggaacgcttgaagacgtatgcccatctcttcgatgacaaggttatgaa

gcagctcaagagaagaagatacaccggatggggaaggctgtcccgcaagcttatcaatggcattagagacaagcaatcagggaagacaatccttgactttttgaagtctgatggcttcgcgaac

aggaattttatgcagctgattcacgatgactcacttactttcaaggaggatatccagaaggctcaagtgtcgggacaaggtgacagtctgcacgagcatatcgccaaccttgcgggatctcctgca

atcaagaagggtattctgcagacagtcaaggttgtggatgagcttgtgaaggtcatgggacggcataagcccgagaacatcgttattgagatggccagagaaaatcagaccacacaaaagggt

cagaagaactcgagggagcgcatgaagcgcatcgaggaaggcattaaggagctggggagtcagatccttaaggagcacccggtggaaaacacgcagttgcaaaatgagaagctctatctgt

actatctgcaaaatggcagggatatgtatgtggaccaggagttggatattaaccgcctctcggattacgacgtcgatcatatcgttcctcagtccttccttaaggatgacagcattgacaataaggtt

ctcaccaggtccgacaagaaccgcgggaagtccgataatgtgcccagcgaggaagtcgttaagaagatgaagaactactggaggcaacttttgaatgccaagttgatcacacagaggaagttt

gataacctcactaaggccgagcgcggaggtctcagcgaactggacaaggcgggcttcattaagcggcaactggttgagactagacagatcacgaagcacgtggcgcagattctcgattcacg

catgaacacgaagtacgatgagaatgacaagctgatccgggaagtgaaggtcatcaccttgaagtcaaagctcgtttctgacttcaggaaggatttccaattttataaggtgcgcgagatcaaca

attatcaccatgctcatgacgcatacctcaacgctgtggtcggaacagcattgattaagaagtacccgaagctcgagtccgaattcgtgtacggtgactataaggtttacgatgtgcgcaagatgat

cgccaagtcagagcaggaaattggcaaggccactgcgaagtatttcttttactctaacattatgaatttctttaagactgagatcacgctggctaatggcgaaatccggaagagaccacttattgag

accaacggcgagacaggggaaatcgtgtgggacaaggggagggatttcgccacagtccgcaaggttctctctatgcctcaagtgaatattgtcaagaagactgaagtccagacgggcgggtt

ctcaaaggaatctattctgcccaagcggaactcggataagcttatcgccagaaagaaggactgggacccgaagaagtatggaggtttcgactcaccaacggtggcttactctgtcctggttgtgg

caaaggtggagaagggaaagtcaaagaagctcaagtctgtcaaggagctcctgggtatcaccattatggagaggtccagcttcgaaaagaatccgatcgattttctcgaggcgaagggatata

aggaagtgaagaaggacctgatcattaagcttccaaagtacagtcttttcgagttggaaaacggcaggaagcgcatgttggcttccgcaggagagctccagaagggtaacgagcttgctttgcc

gtccaagtatgtgaacttcctctatctggcatcccactacgagaagctcaagggcagcccagaggataacgaacagaagcaactgtttgtggagcaacacaagcattatcttgacgagatcattg

aacagatttcggagttcagtaagcgcgtcatcctcgccgacgcgaatttggataaggttctctcagcctacaacaagcaccgggacaagcctatcagagagcaggcggaaaatatcattcatctc

ttcaccctgacaaaccttggggctcccgctgcattcaagtattttgacactacgattgatcggaagagatacacttctacgaaggaggtgctggatgcaacccttatccaccaatcgattactggcc

tctacgagacgcggatcgacttgagtcagctcggggggggataagagaccagcggcaaccaagaaggcaggacaagcgaagaagaagaagtag

***Arabidopsis U6* promoter**

ttcgttgaacaacggaaactcgacttgcctttccgcacaatacatcatttcttcttagcttttttttcttcttcttcgttcatacagtttttttttgtttatcagcttacattttcttgaaccgtagctttcgttttcttctt

tttaactttccattcggagtttttgtatcttgtttcatagtttgtcccaggattagaatgattaggcatcgaaccttcaagaatttgattgaataaaacatcttcattcttaagatatgaagataatcttcaaaa

ggcccctgggaatctgaaagaagagaagcaggcccatttatatgggaaagaacaatagtatttcttatataggcccatttaagttgaaaacaatcttcaaaagtcccacatcgcttagataagaaaa

cgaagctgagtttatatacagctagagtcgaagtagtgattg

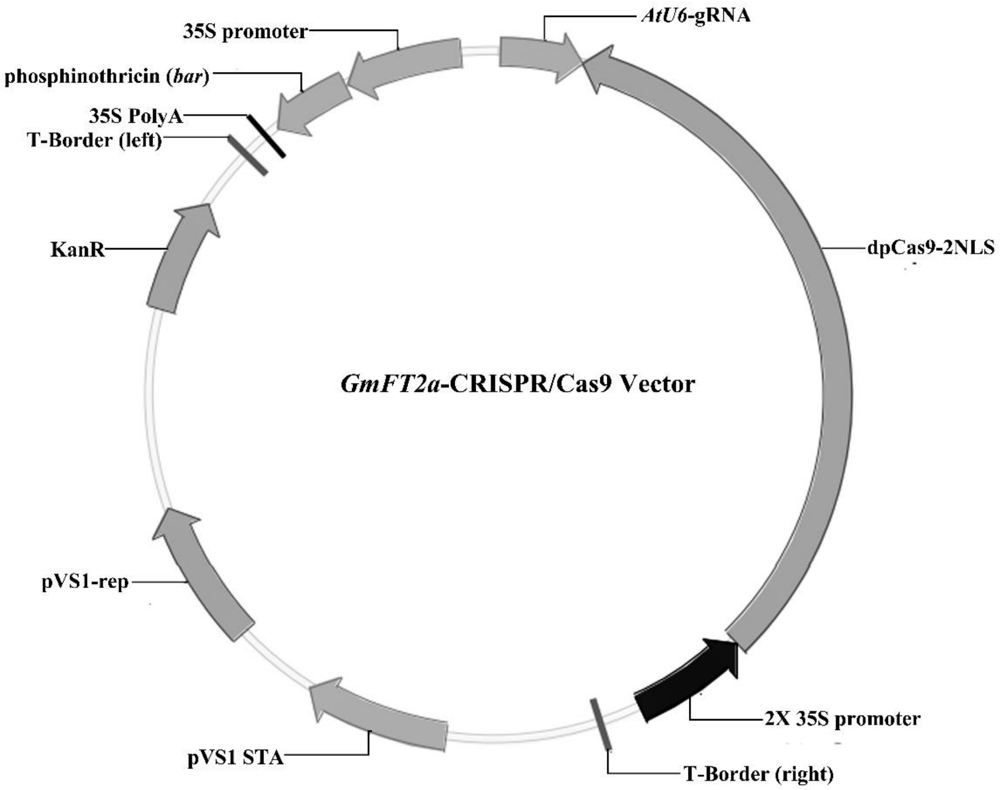

Figure S2 Schematic illustrating the basic architecture of the constructs used for CRISPR/Cas9-mediated genome editing. *AtU6*, *Arabidopsis U6* promoter. gRNA, guide-RNA. dpCas9, Cas9 codon-optimized for dicotyledons. NLS, nuclear localization sequence. The *bar* gene driven by a CaMV 35S promoter is used as a screening marker. KanR, kanamycin resistance gene. pVS1-rep, pVS1 replication origin. pVS1 STA, pVS1 stability function.

Table S3 Primer sequences used in the present study

Primer name	Primer sequence	Size (bp)	Purpose
GmFT2a-F	ATTCATAACAAAGCAAACGAG	653	To amplify the regions which span the target sites
GmFT2a-R	ACTTGACCTTCCCTTAAACAC		
OFF1-F	CGGAGACAAAATGCAGCAGG	684	To examine potential off-target site 1
OFF1-R	GGCCTACAACTATGCTCCCC		
OFF2-F	GGGTCATGCAGAAACTCACCT	533	To examine potential off-target site 2
OFF2-R	TGTGACGTTTGCATGTCTTTTT		
OFF3-F	GGGAAGAGTTTCGGGTCGTT	608	To examine potential off-target site 3
OFF3-R	AAGGTTTCTCATGGTCGGTGT		
OFF4-F	GGCATCATTGCCCCATTTGTT	426	To examine potential off-target site 4
OFF4-R	AACCAAGGAATCAAGTGAAAGCAT		
OFF5-F	AAATTTTATGGCACCACTACCTGC	701	To examine potential off-target site 5
OFF5-R	CTCATCACCATCGCCATCTCT		
OFF6-F	GGGTCATGCAGAAACTCACCT	533	To examine potential off-target site 6
OFF6-R	TGTGACGTTTGCATGTCTTTTT		
Cas9-F	TTGGGGCTCACACCAAACTT	910	To amplify part of the Cas9 coding sequence
Cas9-R	CGATCGCCTTCTTTTGCTCG		
sgRNA-F	AAAAGGCCCCTGGGAATCTG	593	To amplify the region from the *AtU6* promoter to downstream vector sequence spanning the sgRNA
sgRNA-R	GATGAAGTGGACGGAAGGAAGGAG		
GmActin-F	CGGTGGTTCTATCTTGGCATC	249	To amplify *GmActin* as a normalization control.
GmActin-R	GTCTTTCGCTTCAATAACCCTA		
q*GmFT2a*-F	GGATTGCCAGTTGCTGCTGT	160	qRT-PCR analysis (*GmFT2a*)
q*GmFT2a*-R	GAGTGTGGGAGATTGCCAAT		
q*GmActin*-F	CGGTGGTTCTATCTTGGCATC	249	qRT-PCR analysis (*GmActin*)
q*GmActin*-R	GTCTTTCGCTTCAATAACCCTA		
